# Recent advances in the feeding and nutrition of dairy goats

**DOI:** 10.5713/ajas.19.0255

**Published:** 2019-06-26

**Authors:** Arthur Louis Goetsch

**Affiliations:** 1 American Institute for Goat Research, P. O. Box 730, Langston University, Langston, OK 73050, USA

**Keywords:** Dairy Goats, Feeding, Nutrition, Production System, Diet Composition

## Abstract

There have been recent advances concerning research of the feeding and nutrition of dairy goats in a wide array of areas. Ruminally emitted methane and supplementary feedstuffs to a lesser extent make appreciable contributions to the carbon footprint of dairy goats, with the former affected by type of production system and associated dietary characteristics. Unique behavior of goats necessitates careful consideration of the nature of confinement facilities to achieve optimal production by animals differing in social hierarchy. Physiological conditions such as nutritional needs and perhaps health status may influence diet selection by goats in both grazing and confinement settings. Some research suggests that low concentrations of protein and fat in milk of high-yielding dairy goat breeds could involve the type and nature of dietary ingredients as influencing end products of ruminal fermentation. With the relationship between milk urea nitrogen concentration and efficiency of dietary protein utilization, through future research the measure may be a useful tool for diet formulation as in dairy cattle. Effects of dietary inclusion of sources of fats and oils vary considerably depending on their nature, as is also true for byproduct feedstuffs and conventional ones being substituted for. Supplementation of dairy goats with sources of polyunsaturated fatty acids can affect oxidative stress and various feedstuffs influence antioxidant status; however, research addressing the significance of such changes under practical production settings would be beneficial.

## INTRODUCTION

Dairy goats are raised under a wide array of conditions; therefore, studies concerning their feeding and nutrition have addressed a large variety of topics. Ones receiving attention include production system considerations of the carbon footprint, effects of confinement conditions on behavior, stress, and productivity, and factors influencing diet selectivity. Diet composition aspects investigated are effects of concentrate and forage levels on yield and composition of milk and milk products, dietary protein characteristics, influences of the inclusion of different sources of fats and oils, and usage of low-cost and abundant byproducts. Moreover, an increasing number of experiments have addressed stress conditions, including heat and dietary addition of feedstuffs eliciting oxidative stress or improving antioxidant status. The objective of this review is to highlight some of the recent studies conducted in these areas. In this regard, recently there were two related summaries of dairy goat feeding and nutrition research presented at the 3rd and 4th Asian-Australasian Dairy Goat Conferences [1,2]. Although some of the same general areas are naturally addressed, care was taken to avoid review of the same studies and to direct attention to some other areas.

## PRODUCTION SYSTEM

### Methane emission and carbon footprint

Enteric methane emission by ruminants makes a considerable contribution to the carbon footprint of various livestock production systems [3], although few estimates for goats are available. Based on fat- and protein-corrected milk yield, Robertson et al [4] found that 26% to 43% and 48% to 56% of the carbon footprint of a small number of indoor and outdoor New Zealand dairy goat farms, respectively, was attributable to enteric methane emission. The lesser contribution of methane to the footprint and greater variability for indoor vs outdoor production probably relate to higher and more variable levels of concentrate in diets of goats reared indoors. Another sizeable source of carbon was supplementary feed, contributing 13% to 50% of the total emission. This large range in values relates to the dietary level and type of feedstuff, with emission factors (kg of CO_2_-equivalents per ton) ranging from 0 for brewers grain (not considering transportation) and 190 for corn grain to 734, 800, and 940 for avon (byproduct of corn for manufacture of glucose/starch), palm kernel expeller, and cow milk, respectively. Perhaps surprisingly, the carbon footprint relative to fat- and protein-corrected milk production was only numerically lower for indoor vs outdoor farms and similar to New Zealand dairy cattle farms. The carbon footprint on a product basis is usually lower for more intensive than extensive production systems because of generally greater dietary levels of concentrate, use of breeds highly selected for level and efficiency of production, and less energy expended in the act of grazing [5]. Conversely, on a land area basis, the footprint is normally higher for intensive vs extensive production systems primarily because of considerable use of harvested feedstuffs, notably concentrates. In accordance, Robertson et al [4] found a land area carbon footprint approximately twice as great on indoor vs outdoor farms. However, these values do not take into account soil carbon sequestration, which substantially impacts the carbon footprint of extensive and semi-intensive production systems where most nutrients are derived via grazing [5].

### Confinement conditions

Due to the common behaviors of goats and strength of their social hierarchy, feeding and feed access conditions may be relatively more important than for cattle and sheep. This was studied by Jørgensen et al [6] with mid-lactation Norwegian dairy goats given access to hay of an unknown type or grass silage. Animals were in groups of six in 3×1.9 m pens subjected to treatments of 1, 2, or 3 goats per feeding space. Time spent eating decreased and that waiting for feeder access increased with the increasing number of goats per space, which was accompanied by decreasing group intake of silage. Aggressive interactions increased with the increasing number of goats per space, to a greater extent for hay. This, along with no treatment effect on hay intake, suggest a greater preference for hay. Although, the reported neutral detergent fiber (NDF) concentration in offered silage of 8.9% may question the nature of the silage used and application of the findings to other conditions. Nonetheless, because intake was measured on a group basis rather than for individual animals and there was greater variation in feeding time among individuals for hay, it is likely that intake of hay varied more among animals than that of silage. Some of the effect of more than one animal per space on time spent feeding was due to more dominant animals preventing feeder access by ones of lower social rank even when the feeder was not being used, which may be more likely than if the pen space allowance had been greater than 0.9 m^2^/animal. Moreover, an outside run area such as used by Stachowicz et al [7] could be useful in this regard.

The study of Stachowicz et al [7] was with 13 dairy goat farms in Switzerland and Germany. The quality of indoor areas and outside runs varied substantially, with differences in space allowance, ease of access to the outside run, partitions for visual cover, lying niches, additional hayracks, areas for climbing, presence and type of enrichment items, weather protection, etc. Goats used the outside run regardless of quality of the inside area. But, use of the outside run rose as quality of its conditions increased, which was manifested through a longer length of stay outside rather than a greater number of visits. All outside enrichment items were used when multiple ones were present, with most time spent lying and less agonistic social interactions. Although neither feed intake nor productivity were assessed, differences in behavior observed were suggested to have potential economic impact as well as animal welfare considerations. The most important aspect of the indoor area was to have a high space allowance and items available for attention to limit agonistic behavior and increase time spent lying without interruption.

In addition to overall pen design and feeder availability, the nature of feeders and associated management practices are important to realize high levels and efficiencies of production by all animals in a group regardless of characteristics such as size, age, presence or horns, and social rank. The study of Hillmann et al [8] with Swiss dairy goats characterized differences in behavior of horned and hornless animals in separate pens. It was stated that horned goats interact more through visual threats with a larger and more respected individual distance compared with hornless goats that incur greater physical interaction during more frequent conflict. Feeding station treatments were head restraint for 1 hour after the two daily meals and a physical feed barrier between heads when feeding. The partition was solid but not large enough to prevent some attempted interaction between adjacent animals. Head restraint allowed low-ranking animals to feed longer than ones unrestrained, particularly for horned animals. But, restraint necessitated the presence of the partition to limit agonistic activities. Without restraint, middle- and low-ranking hornless goats used feeders relatively more than did horned goats but with considerable agonistic behavior and physical contact, because of little impact of the individual-animal distance, which limited total feeding time. Thus, restraint increased feeding time of low-ranking hornless goats and all horned goats. Body weight (BW) did not differ among treatments during the 5 to 6 week study, but effects on daily feed intake are unclear and the goats were nonlactating.

The experiment of Nordmann et al [9] was similar to that of Hillmann et al [8] in some respects. Pregnant German Improved Fawn goats were used in groups of 36 in pens with or without a feeder barrier (i.e., head partition), but with feeding behavior observed over 48 hours rather than 1-hour periods. The activity of high-ranking goats was not noticeably affected by the partition, although an increased body condition score determined at the lumbar spine was postulated to have resulted from more continuous feed consumption with less interruption by agonistic actions being imparted to lower-ranking animals. It was postulated that low-ranking goats were at feeders with a partition less during peak feeding hours because lack of visual contact prevented knowledge of the location of higher-ranking animals and, thus, if an aggressive encounter was likely. An effect such as this can influence the potential for diet selectivity depending on the amount of feed offered relative to that consumed, the number of meals, etc. The lesser amount of time spent feeding over the 2-day period by middle-ranking animals was suggested to have resulted from fewer displacements and a greater rate of feed intake. With the potential influence of eating time on efficiency of energy utilization [10–12], an effect such as this could influence the efficiency of production of lactating animals.

### Grazing settings

Goats have considerable ability to select different plants and plant parts compared with cattle and sheep, with the botanical composition of the diet more reflective of the array of species available [13]. Moreover, the physiological state of the animal, as impacting nutrient and energy needs, can influence selectivity and diet composition. As an example, with Creole goats in a woodland area of the central Monte Desert in Argentina, Egea et al [14] observed not only greater dry matter (DM) intake by goats in early lactation compared with nonlactating animals but also relatively more consumption of high-protein and tanniferous shrubs. The latter findings presumably relate to the greater protein requirement of goats when lactating than dry and tannin binding of protein in the rumen to increase intestinal amino acid absorption. Moreover, the metabolizable energy (ME) concentration in the diet selected by lactating goats tended to be greater, and though not measured, it was suggested that this resulted from increased grazing time and rate of biting. The study of Askar et al [15] with Boer goats grazing grass-forb pastures supports involvement of rate of biting, in that botanical and chemical composition of the diet selected by lactating does, growing wethers, and yearling wethers were similar, but the rate of ME intake by lactating does was greatest. Time spent grazing was similar among animal types, which may relate to a likely increase in heat energy associated with longer grazing time and little or no association between rate of biting and energy expenditure [15–17]. Some findings of Manousidis et al [18] with adult Greek goats grazing Mediterranean woody rangeland over a 2-year period are similar to those of Egea et al [14]. An analysis was conducted to determine chemical characteristics responsible for the selection of specific plants and plant groups and influence of animal physiological stage. The crude protein (CP) concentration in plants had a greater influence on selection in spring and summer when goats were lactating compared with the autumn, although confounding of physiological state and season is a consideration.

Fedele et al [19] noted considerable change in the ingredient and chemical composition of the diet of Maltese goats of Italy from pregnancy through lactation with free access to alfalfa hay, pasture hay, flaked barley, chickpea, broad beans, and beet pulp. Overall, the CP concentration increased with advancing pregnancy and decreased as days-in-milk (DIM) decreased, the latter concomitant with an increased level of starch. Intake of DM peaked in early/mid-lactation for goats both given free access to the different feedstuffs and ones managed traditionally. Feedstuffs being consumed by free-choice goats varied throughout the study; however, the diet level of NDF was steady at approximately 40%. Although the 16-week study of Goetsch et al [20] was with growing Alpine doelings and there were considerable differences in other conditions, the findings are not in close accordance with those of Fedele et al [19]. A concentrate mixture of 72.8% ground corn, 15.2% soybean meal, 6% fish meal, and 6% dried molasses product was offered at 25%, 50%, or 75% of the diet (i.e., 25C, 50C, and 75C, respectively) with wheat hay comprising the other portion and a feeding rate of 105% to 110% of previous consumption. There was also a treatment with free access to concentrate and forage (FC). The dietary level of concentrate was 26.3%, 53.2%, 79.8%, and 83.6%, DM intake was 626, 641, 623, and 704 g/d, and average daily gain (ADG) was 53, 71, 81, and 105 g for 25C, 50C, 75C, and FC, respectively. Favorable effects of the FC treatment on ADG and ADG:DM intake were manifested in the last 8 weeks of the experiment, which may relate to adaptation to a relatively low dietary level of NDF (i.e., <20%) as well as other factors such as increasing capacity for lipid accretion with advancing time.

Consumption by ruminants of feedstuffs containing phenolic compounds can have a variety of effects in addition to ruminal protein binding. One addressed recently by Chávez-Servín et al [21] is that ingested phenolic compounds can be present in milk, with potential benefits to human health in part relating to change in antioxidant capacity. The study addressed the effects of type of production system and season (dry vs wet) in Mexico on phenolics in goat milk, whey, and cheese. Alpine does at 60 DIM were in a free-range grazing setting or confined with consumption of alfalfa hay and concentrate. Phenolic compound levels were greater for the semi-intensive vs intensive system and in the dry vs wet season, and antioxidant capacity was greater in the dry season and in some cases for grazing vs confinement. Plant species were only described in a general manner, and differences presumably relate to the level of phenolics in particular plants present. Moreover, additional production variables such as milk yield and levels of other constituents could have been influenced, as other conditions sometimes altered by phenolics include ruminal methane emission [22] and biohydrogenation of fatty acids (FA) that could affect bioactive conjugated linoleic acid (CLA) isomers reaching the mammary gland to potentially lessen de novo FA synthesis.

## DIET COMPOSITION

### Concentrate and forage

The nature of the diet influences not only the level of milk produced by dairy goats but also its composition, with an impact on yield and quality of products, namely cheese. In this regard, through a survey of dairy goat farms in Italy Sandrucci et al [23] addressed factors contributing to ‘fat/protein reversion’, which is when the level of fat drops below that of protein to adversely affect cheese yield and sensory quality. It was proposed that relatively low dietary levels of forage and ether extract and high somatic cell count were contributing factors. Relatedly, breeds of dairy goats with greatest milk yield, namely Alpine and Saanen that were reared on most farms surveyed by Sandrucci et al [23], have low milk concentrations of fat and protein relative to other breeds [24]. Although responsible factors are unclear, from publications describing common feeding practices on farms of dairy cattle [25,26] and dairy goats [23,27], as well as reviews of recent research of the feeding management practices of dairy goats [1,2,24], some possibilities become apparent. Dairy cattle are usually fed diets higher in high-quality forage and lower in concentrate than dairy goats. Adequate effective fiber in dairy goat diets is often achieved by inclusion of low levels of very low-quality fibrous roughages [e.g., 28–34]. High dietary levels of byproduct feedstuffs with fiber highly degradable in the rumen may be more common on dairy cattle than goat farms. Lastly, in many instances the legume alfalfa is at higher levels in dairy goat diets [e.g., 28–35] relative to rations of dairy cattle [e.g., 26,27]. These differing conditions could result in a more ‘grain-’ than ‘forage-like’ array of digestion endproducts (i.e., low vs high acetate:propionate) that contributes to low fat and protein concentrations in milk of high-yielding dairy goat breeds.

Although research to specifically address the postulate put forward above is needed, there may be some support in the study described by Inglingstad et al [36] and Steinshamn et al [37] and that of Monzón-Gil et al [38]. In the former experiment, milk of Norwegian goats grazing two types of pasture was higher in protein concentration, and generally higher in fat as well, than milk from goats consuming diets based on hay of low quality. Inglingstad et al [36] suggested that this related to a small difference in dietary level of concentrate, but levels varied only by 6.3 percentage units (i.e., 43.5% vs 37.2%) and concentrate was not high in starch, consisting of 27.8% barley, 26.3% oats, 15.9% wheat bran, 6.5% molasses, 6.0% sugar beet pulp, and 17.5% other ingredients. Monzón-Gil et al [38] used Canarian Majorera yearling doelings in two 41-week lactations to compare total mixed ration (TMR) and separate concentrate and forage (SR) feeding systems. Ryegrass hay was the forage fed at 15% of the diet, and the concentrate mixture consisted of 33% corn grain, 26.5% dehydrated alfalfa, 24% dehydrated beet pulp, 10% wheat bran, and 6.5% soybean meal. However, individual versus group housing was not specified. The TMR treatment resulted in increases of 8% to 9%, 42% to 44%, 10%, 5%, and 15% in concentrate intake, forage intake, milk yield, milk concentrations of protein and fat, and yields of protein and fat, respectively. Though not of the Alpine or Saanen breed, average milk yield with one daily milking was 1.45 and 1.85 liters for TMR and 1.29 and 1.69 liters for SR in the first and second lactations, respectively. Beneficial effects of the TMR treatment were thought due to both the increased dietary level of forage and simultaneous intake of forage and concentrate that improved fiber fermentation and increased microbial production of acetic acid.

As alluded to above, in some cases dairy goats are fed diets with high levels of concentrate that can result in acidotic conditions in the rumen, acute or more commonly subacute (SARA). In a review of recent literature in this area, Giger-Reverdin [39] noted that one of the most important factors influencing susceptibility of individual animals to SARA is the temporal patterns of feed consumption and rumination. Animals consuming a high proportion of feed immediately after it is offered are relatively more prone to SARA than others ingesting smaller amounts with intermittent rumination bouts. Moreover, there is tremendous variability in feeding and rumination behavior both among animals and over days within animals. However, as perhaps an example of ‘nutritional wisdom’, it was noted that dairy goats can modify feeding behavior when given a high concentrate diet to lessen susceptibility to SARA, and also that selection of more or less fibrous feed particles may shift over time. This may be relevant to findings of Goetsch et al [20] noted earlier with separate access to concentrate and forage. Consumption of feedstuffs with condensed tannins by animals infected with internal parasites could be viewed as nutritional wisdom or perhaps more appropriately ‘self-medication’ [40]. Moreover, although not evaluated in a cafeteria-like feedstuff choice experiment, Muklada et al [41] reported the potential for consumption of willow with salicin to provide anti-inflammatory activity and lessen somatic cell count in lactating dairy goats. However, it was noted that salicin may limit the voluntary intake of willow.

### Protein

The concentration of urea in milk (MUN) is commonly used to evaluate the efficiency of dietary CP utilization by dairy cattle [42,43]. The level is generally greater in the milk of dairy goats than cattle. Target MUN levels in dairy cattle are based on predictions when animals consume diets formulated according to accepted nutrient requirement recommendations. Levels less than targets indicate a deficiency of amino acid absorption and greater values reflect inefficient nitrogen usage, economic loss, and nitrogen loading into the environment [43].

Usefulness of MUN for dairy goats has not been extensively studied. However, recently Rapetti et al [44] addressed this with data compiled from five studies with Saanen goats. The relationship between MUN and dietary CP concentration was improved slightly by including the non-fiber carbohydrate concentration in the regression equation because of its influence on microbial capture of ruminally degradable CP. The average dietary CP concentration for the database was 16.3%, which is in close accordance with requirements based on Nsahlai et al [45] and NRC [17]. With average database values for BW, DM intake, milk yield, and milk protein concentration and use of the Langston Interactive Nutrient Calculation system (LINC; http://www.luresext.edu/?q=Nutrient-Calculators), requirements were 11.2% metabolizable protein and 16.6%, 15.9%, and 15.2% CP with rumen undegraded protein levels of 20%, 40%, and 60% of CP, respectively. The average MUN in the study of Rapetti et al [44] was 34.2 mg/dL, with values ranging from 11.9 to 67.5 mg/dL. The optimal range suggested by Brun-Bellut [46], at which efficiency of usage of ruminally available nitrogen is high, is 28 to 32 mg/dL. This range is approximately twice common target levels for dairy cattle [42]. Some diets of Rapetti et al [44] had dietary CP levels above needs based on the intercept (i.e., 22.9 mg/dL) of the regression of MUN on balance of protein digestible in the intestine with microbial growth limited by ruminally available nitrogen (PDIN; balance equal to the difference between the requirement and supply as a percentage). However, the equation of the regression of MUN against dietary CP concentration results in a dietary CP concentration of 14.1% when the MUN is 22.9 mg/dL and PDIN is 0% (i.e., average supply equal to the requirement). The concentration of 14.1% CP is less than the database average, but, there is variability among individual animals in nutrient and energy needs. Thus, it may be germane to consider that many nutrient requirement recommendations include what might be viewed as a safety factor so that requirements of most or all animals are met rather than only that of the average animal and ones with lesser needs. Clearly, future research attention should be given to use of MUN as a diet composition management tool for dairy goats as well established for dairy cattle.

### Fats and oils

Effects of dietary fat and oil supplementation on milk yield by dairy goats are more likely in early lactation than later presumably because of differences in DIM at which DM intake and milk yield peak [47]. Moreover, Ferlay et al [48] summarized that dietary inclusion of fats and oils more frequently increases the concentration of fat in milk of dairy goats than dairy cattle [e.g., 28, 31, 49 with whole linseed]. Although, there are many instances when this has not occurred, examples being studies of Shi et al [50] with safflower seed oil and the microencapsulated fish oil product treatment of Caroprese et al [49]. Disparate effects are because of differences in the nature, composition, and level of the specific supplemental lipid ingredient(s) as well as other characteristics of the diet influencing level of feed intake, ruminal digestibility, FA binding to digesta, completeness of biohydrogenation, etc. Lastly, effects of the FA profile in specific feedstuffs and plants being consumed, such as forages vs. concentrates and plant species, on the FA composition of milk fat of goats are generally similar to those in cattle apart from differences relating to the quantity of bioactive CLA isomers reaching the mammary gland. The study of Caroprese et al [49] provides an example of considerable improvement in the FA composition of milk of goats through lipid supplementation. Feeding 150 g DM/d of whole linseed to Garganica goats of Italy (44 kg BW) at 61 DIM in 1.5 kg DM/d of supplemental concentrate markedly reduced anthrogenic and thrombogenic indices of milk. However, it is unclear if similar findings would occur with a higher yielding goat breed as well as with lower concentrate and greater forage consumption.

In a review of literature concerning the production of *trans* and conjugated FA in dairy ruminants, Ferlay et al [48] included comparisons of dairy cattle and goats when adequate research existed. One major difference is that goats are much less prone to milk fat depression than dairy cattle. There is less impact of bioactive CLA isomers on the activity of mammary gland enzymes of goats synthesizing FA de novo. Moreover, the activity of ruminal microbes involved in biohydrogenation is affected by dietary levels of concentrate with supplemental lipid less than in cattle. Higher feed intake by goats relative to BW minimizes ruminal digesta residence time that can influence the extent of biohydrogenation, and this also may limit the effects of ingested plant secondary metabolites on biohydrogenation. Also, goats seem less prone to negative effects on DM intake in early lactation compared with dairy cattle that occur via elevated blood levels of non-esterified FAs resulting from high dietary fat levels coupled with tissue mobilization [28,51].

### Byproducts

With the increasing cost of conventional feedstuffs, many agricultural and industrial co- or byproducts, as well as various novel materials, continue to be evaluated as ruminant feedstuffs. The recent study of Arco-Pérez et al [52] provides an example of how byproducts can be used effectively in diets of lactating dairy goats when included at low levels. Olive byproduct silage and tomato surplus silage replaced oat hay at 20% without deleterious effects on performance, and long-term feeding of tomato silage increased BW gain. The design of the study of Fernández et al [53] was somewhat different, however, with pelleted orange leaves totally replacing pelleted alfalfa at 45% of the diet. Forage and concentrate were fed separately, resulting in concentrate levels of 65.1% and 69.1% and NDF in consumed DM of 31.5% and 26.2% for the alfalfa and orange leaves diets, respectively. Total DM intake was less for the diet with orange leaves (1.36 vs 1.61 kg/d), leading to higher DM digestibility (71.1% vs 63.5%) and intake of digested DM (1.07 and 0.97 kg/d for the alfalfa and orange leaves diets, respectively). But, milk yield was similar between treatments (1.33 and 1.25 kg/d), so that yield:DM intake was greater for the orange leaves diet (0.92 vs 0.79). In addition to the differences in dietary concentrate level and digestibility that partially compensated for lower DM intake of the orange leaves diet, less energy loss in ruminally emitted methane (18.1 and 12.3 g/d for the alfalfa and orange leaves diets, respectively) probably contributed to more efficient use of DM of the orange leaves diet. Although essential oils in orange leaves may have contributed to the difference in methane, it seems likely that varying dietary levels of concentrate and fiber had an impact as well.

The recent study of Sousa et al [54] with forage palm was in regard to the considerable amount of this byproduct available in some areas and few alternative uses. Dairy goats of an unspecified breed at 30 DIM were fed diets with 42.1%, 35.0%, 29.1%, 23.6%, and 19.9% forage palm and bermudagrass hay at 16.0%, 24.4%, 31.6%, 38.3%, and 42.8%, respectively. Levels were varied based on forage palm being low in NDF (e.g., stated to be typically 20% but analyzed at 34%) and high in minerals, particularly oxalates. There was a substantial decrease in DM intake (1.98 to 1.19 kg/d) as the level of bermudagrass hay increased and that of forage palm decreased, which corresponded to a smaller increase in DM digestibility (71.0% to 82.5%) presumably due to increased digesta residence time in the rumen. Nonetheless, with ME intake of 22.3, 19.6, 18.8, 17.5, and 15.5 MJ/d, milk yield was 1.52, 1.58, 1.57, 1.67, and 1.67 kg/d that resulted in a substantial increase in the ratio of milk yield to DM intake (i.e., 0.78, 0.93, 1.06, 1.20, and 1.49 for forage palm levels of 42.1%, 35.0%, 29.1%, 23.6%, and 19.9%, respectively). Hence, in some manner, the moderate to high dietary levels of forage palm had a very adverse effect on the efficiency of nutrient utilization for milk production though not on feed intake. Dietary level of NDF would not seem to have been involved, varying only from 31% to 42%. One possible factor is oxalate in palm, although the level was not analyzed, and the authors suggested that adverse effects were unlikely with these dietary inclusion levels. Hence, further research with this byproduct is warranted.

Another byproduct continuing to receive research attention in various settings is crude glycerin of the biodiesel industry. One factor influencing its use in ruminant diets is purity, although effects can be minimized by limiting dietary levels. For example, Novais-Eiras et al [55] included crude glycerin of low purity (43.4% glycerol, 2.6% methanol, and 23.8% FA) at 0%, 3%, 6%, and 9% of concentrate supplemented to Alpine goats in mid-lactation for total dietary crude glycerin levels of 0%, 1.34%, 3.05%, and 3.57%, respectively. No adverse effects on feed intake, digestibility, or milk yield were observed, and milk fat increased with increasing inclusion level. There were some favorable changes in milk FA composition in regard to human health effects, and crude glycerin served as an important source of glucose early after feeding and improved nitrogen retention as well.

## STRESS CONDITIONS

As mentioned above, diets of lactating goats as well as cattle often are supplemented with polyunsaturated FA that can increase milk fat concentration and(or) improve the FA profile of milk and milk products for beneficial effects on human health. However, because of potential oxidation, polyunsaturated FA supplementation can increase oxidative stress, leading to the study of Mavrommatis et al [56] with the microalgae *Schizachy trium* sp. to determine its optimal dietary level. In addition to measures such as feed intake and milk yield and concentrations of fat, protein, and specific FA, antioxidant activity and extent of oxidative damage were characterized. Dietary concentrate consumed by Alpine×local Greek goats at 150 DIM included a commercial product to achieve daily microalgae intake of 0, 20, 40, or 60 g. But, the 60 g treatment decreased concentrate intake most likely a consequence of the smell and(or) palatability of the product, resulting in microalgae intake of 40 g/d. Milk fat concentration was markedly decreased by the 40 and 60 g treatments, presumably because of adverse effects of some of the polyunsaturated FA on the activity of fibrolytic bacteria and, thus, decreased acetate production and concomitant de novo FA synthesis in the mammary gland. Although, there would also seem potential for effect of bioactive CLA isomers reaching the mammary gland because of decreased ruminal biohydrogenation. Based on changes in indicators of antioxidant activity (e.g., superoxide dismutase, glutathione transferase, glutathione reductase, and ferric reducing ability of plasma) and of oxidative stress realized (catalase and malondialdehyde) in blood and milk, the 20 g level appeared optimal. But, milk fat concentration was numerically 0.53 percentage units less than for the control treatment (i.e., 2.97% vs 3.50%). Even though there is considerable interest in oxidative stress as affected by dietary and environmental conditions because of the potential impact on immunity, health, and longevity of dairy animals, it is difficult for such factors to be considered in typical relatively short-term experiments to determine practical and economic implications.

Somewhat converse to the experiment of Mavrommatis et al [56], one postulate of the experiment of Caroprese et al [49] was that dietary supplementation with sources of FA can enhance cell-mediated and humoral immune responses of dairy goats. Supportive studies cited appeared primarily with other species and stressful conditions such as from heat exposure. The study involved Garganica goats of Italy at 60 DIM and 44 kg BW grazing pasture but also given approximately 1.5 kg DM/d of concentrate. Neither supplementation with 50 g DM/d of microencapsulated fish oil nor 150 g DM/d of whole linseed affected cell-mediated immunity. However, whole linseed supplementation had a number of effects indicating lower humoral responses. The different than expected findings were suggested to involve the lack of stressful conditions. Conversely, considerable favorable effects of whole linseed on the FA composition of milk in regard to human health attributes (e.g., increased polyunsaturated and decreased saturated FA) would indicate potential for influence on oxidative stress as noted by Mavrommatis et al [56].

Goetsch [2] overviewed an experiment with lactating dairy goats [57] indicating that diets with an elevated cation-anion difference (DCAD) may be beneficial with heat stress conditions at least in part by increasing water intake. Findings of de Lima et al [58] suggest that dietary inclusion of seaweed *Gracilaria birdiae* may mitigate heat stress in lactating dairy goats in a different manner. Seaweed was included at 0%, 4%, 8%, and 12% of the diet of Saanen goats at 60 DIM. There were improvements in respiration rate, rectal temperature and surface temperature when air temperature was elevated. Heat stress normally elevates the formation of reactive free radicals that can elicit oxidative damage to adversely affect immune function and overall health status. It was proposed that these effects related to inhibition of free radical formation by seaweed antioxidants (e.g., sulfated polysaccharides) that lessened total oxidative stress.

The study of Tian et al [59] concerned effects of the anthocyanin class of flavonoids on variables relating to antioxidant status. Purple corn (*Zea mays* L.) stover silage was included in the diet of multiparous Saanen goats at 75 DIM compared with sticky corn silage. Similar to phenolic compounds addressed by Chávez-Servín et al [21], dietary anthocyanins were found in goat milk, with increased superoxide dismutase in plasma and milk but no differences in other antioxidant measures. Feed intake and milk yield were not different between diets, as was true for milk concentrations of fat and protein. But, even though feed intake was ad libitum, DM intake was only approximately 2.1% BW with milk yield from one milking per day that was 76% greater than DM intake.

## CONCLUSION

Methane emission by dairy goats makes a greater and less variable contribution to the carbon footprint of outdoor vs indoor production systems on a product basis, though such comparisons may vary if land area and soil carbon sequestration are considered. Because of the unique behaviors and a strong social hierarchy of dairy goats, the design of confinement facilities could influence the level and(or) efficiency of productivity. Diet selection of dairy goats can vary with the physiological state, and there is evidence for nutritional wisdom and self-medication warranting further study. Moreover, research is needed to address optimal dietary composition for production of milk of high quality for processing, as is also true for the wide array of byproduct feedstuffs dairy goats will consume. The numerous effects of feedstuffs high in FA vary considerably with factors such as source and level and other dietary characteristics. The inclusion of various dietary ingredients can influence oxidative stress conditions and antioxidant status, but long-term practical influences on production are unclear.
